# The emerging role of tissue regulatory T cells in tissue repair and regeneration

**DOI:** 10.3389/fimmu.2025.1640113

**Published:** 2025-08-29

**Authors:** Abdul Raheem, Ilyas Khan, Iqbal Ahmad, Abdul Wajid, Mohammad Y. Alshahrani, Fuad M. Alzahrani, Khalid J. Alzahrani, Abdul Qadeer, I-Chuang Liao, Chien-Chin Chen

**Affiliations:** ^1^ College of Veterinary Medicine, Huazhong Agricultural University, Wuhan, China; ^2^ Institute of Animal Sciences, Chinese Academy of Agricultural Sciences, Beijing, China; ^3^ State Key Laboratory for Animal Disease Control and Prevention, Harbin Veterinary Research Institute, Chinese Academy of Agricultural Sciences, Harbin, China; ^4^ Faculty of Pharmacy, Gomal University, Dera Ismail Khan, Khyber Pukhtunkhwa, Pakistan; ^5^ Department of Clinical Laboratory Sciences, College of Applied Medical Science, King Khalid University, Abha, Saudi Arabia; ^6^ Department of Clinical Laboratories Sciences, College of Applied Medical Sciences, Taif University, Taif, Saudi Arabia; ^7^ Department of Cell Biology, School of Life Sciences, Central South University, Changsha, China; ^8^ School of Medicine, College of Medicine, National Sun Yat-sen University, Kaohsiung, Taiwan; ^9^ Institute of Clinical Medicine, College of Medicine, National Cheng Kung University, Tainan, Taiwan; ^10^ Department of Pathology, Chi Mei Medical Center, Tainan, Taiwan; ^11^ Department of Pathology, Ditmanson Medical Foundation Chia-Yi Christian Hospital, Chiayi, Taiwan; ^12^ Department of Cosmetic Science, Chia Nan University of Pharmacy and Science, Tainan, Taiwan; ^13^ Doctoral Program in Translational Medicine, National Chung Hsing University, Taichung, Taiwan; ^14^ Department of Biotechnology and Bioindustry Sciences, College of Bioscience and Biotechnology, National Cheng Kung University, Tainan, Taiwan

**Keywords:** regulatory T cells, tissue, injury, repair, regeneration

## Abstract

Regulatory T cells (Tregs) are a unique subset of T cells vital for maintaining immune balance, preventing autoimmune diseases, and controlling immune responses. First identified in the early 1990s, Tregs are now well recognized for their role in suppressing excessive immune reactions and promoting tolerance to the body’s tissues. Among the broader Treg population, Tissue regulatory T cells (Tissue Tregs) are distinct as they do more than suppress immunity; they actively contribute to tissue repair and regeneration. Studies in both mice and humans have highlighted the important role of in aiding tissue repair and maintaining tissue integrity. Recent research reveals that Tregs participate in wound healing and tissue regeneration across various organs, including the heart, liver, kidneys, muscles, lungs, bones, and central nervous system. These discoveries emphasize the wide-ranging and significant influence of Tregs in fostering recovery and healing in different tissues throughout the body. These cells are characterized by their ability to produce a variety of growth factors, cytokines, and signaling molecules that support the repair and regeneration of damaged tissues. In this review, we present an overview of the emerging understanding of Treg-mediated repair processes in damaged tissues and organs.

## Introduction

The worldwide rise in organ dysfunction caused by acute injuries, chronic diseases, or aging is increasing the need for organ transplants. However, the limited availability of donor organs and the use of immunosuppressive drugs pose significant challenges (1), leading to the search for alternative therapies. Recent advances in human pluripotent stem cell research, known for their ability to self-renew and differentiate into various cell types, offer a potentially limitless source of therapeutic cells for transplantation (2, 3). Despite this potential, there is limited clinical evidence of their long-term survival after transplant, possibly due to issues like poor cell viability and ongoing immune rejection (4, 5). Additionally, regenerative therapies focused on natural tissue repair, such as growth factor-based methods, have shown mixed results in clinical trials because of safety and cost concerns (6, 7). Therefore, it is crucial to develop strategies to improve tissue repair and regeneration.

Traditionally, the immune system has been seen mainly as a defense against pathogens, a view that has shaped how it appears in textbooks for over a century (8). However, beyond its protective role, the immune system is now understood as a crucial part of maintaining tissue homeostasis and supporting physiological processes like development, reproduction, wound healing, and tissue regeneration (9). Recent studies have emphasized the important role of innate immunity, especially the different polarization states of macrophages, in coordinating the complex events needed for tissue repair and regeneration (10, 11). Still, new evidence suggests that the adaptive immune system, particularly tissue Tregs, also has a key role in these processes (12, 13).

Forkhead box P3-expressing (Foxp3^+^) Tregs have long been recognized for their role in modulating immune responses and maintaining immune homeostasis (14). These cells, traditionally seen as suppressors of excessive immune activation, are now understood to have non-traditional functions that go beyond immune regulation (15, 16). Recent studies using transgenic mouse models, in which Tregs were specifically removed, have revealed their important roles in influencing non-immunological processes, including tissue repair and regeneration (15, 17). This new evidence indicates that Tregs are crucial in promoting repair and regeneration within injured tissues (18). A particularly intriguing aspect of Treg biology is their organ-specific functionality, with recent studies showing how tissue-resident Tregs respond to injury tailored to each organ’s distinct microenvironment (19, 20). This organ-specific behavior of Tregs highlights their role in tissue repair, not only as suppressors of inflammation but also as active contributors to the regeneration of damaged tissues (9, 11, 21). Treg cells not only suppress immune responses but also promote tissue repair by secreting pro-repair mediators. These factors act directly on resident structural and parenchymal cells within a tissue-specific context (22).

In this review, we will focus on the emerging roles of Tregs in tissue repair and regeneration, emphasizing their ability to modulate inflammation, promote healing, and interact with other immune cells. By exploring recent advancements in our understanding of tissue-resident Tregs and their functions, this review aims to shed light on the complex interplay between tissue Tregs and tissue regeneration, offering insights that could lead to new therapeutic strategies for enhancing tissue repair and restoring function more effectively.

## Treg diversity

Tregs are essential for immune regulation and exhibit considerable diversity. Besides the well-known CD25 marker, they express a range of activation markers, including both co-stimulatory and co-inhibitory molecules such as PD-1, ICOS, LAG-3, CD27, CD69, LAP, and CTLA-4. They can also express members of the TNF receptor superfamily, like GITR and OX40, along with adhesion-related markers such as CD62L and CD49b. Additionally, they possess receptors involved in guiding their migration to peripheral tissues, including CCR7, CCR4, CCR6, CD103, and CCR5 (23). Foxp3 is a key transcription factor primarily expressed in CD4^+^ CD25^+^ Tregs in mice. It is present at minimal levels in CD4^+^ CD25^-^ effector T cells and is mainly absent in CD8^+^ T cells (24, 25). Foxp3 is crucial for Treg development and their capacity to suppress immune responses (24–27). In mice, Tregs are typically identified as CD4^+^ CD25^high^ Foxp3+. However, in humans, not all Foxp3-expressing cells display high CD25 levels (23), nor do they all possess immunosuppressive functions (28).

In humans, the expression of the IL-7 receptor alpha chain (CD127) is inversely related to Foxp3 expression. CD4^+^ CD127^low^ T cells in humans exhibit similar suppressive functions to CD4^+^ CD25^high^ T cells observed in laboratory settings (29). Therefore, human Tregs are more accurately defined as CD4^+^ CD25^high^ CD127^low/-^ Foxp3+. While this constitutes the core phenotype of Tregs, studies utilizing flow cytometry and RNA sequencing have demonstrated that both human and mouse Tregs are highly diverse (30). Their origin can also be used to categorize Tregs. Thymic Tregs (tTregs) are derived from CD4^+^ CD25^high^ thymocytes that undergo positive selection after their T-cell receptors (TCRs) engage with high-affinity self-peptides presented by antigen-presenting cells and medullary thymic epithelial cells on MHC class II molecules. This process, combined with signals from cytokines such as IL-2 or IL-15 and TGF-β, promotes the differentiation of these cells into fully committed tTregs (31, 32). Peripheral Tregs (pTregs) develop from naive CD4^+^ Foxp3- T cells in secondary lymphoid organs and peripheral tissues, in response to cytokines like TGF-β upon encountering antigens (33).

Moreover, Tregs can be further classified by their differentiation stage (e.g., naive Tregs marked by CD45RA^+^ or activated Tregs) and their activation status (e.g., quiescent central Tregs characterized by CD44^low^ CD62L^high^ or effector Tregs with CD44^high^ CD62L^low^). They can also be identified based on the expression of transcription factors that define specific T helper cell lineages, such as T-bet for Th1, Gata3 for Th2, and RoRγt for Th17 (30, 34). In recent years, the role of Tregs localized in non-lymphoid tissues has garnered increasing interest, extending beyond their traditional immune-suppressive functions. Tregs are present in various tissues under steady-state conditions and often accumulate following tissue injury. They are essential for maintaining tissue homeostasis and supporting repair through interactions with local tissue cells. These Tregs, called “tissue Tregs,’ share common features across different tissues but also display tissue-specific characteristics that suit the needs of each environment (35). Tissue-resident Tregs possess a limited T cell receptor (TCR) repertoire, likely recognizing self-antigens unique to the tissues they inhabit, such as fat tissue, muscle tissue, skin, lung tissue, intestines, and other non-lymphoid tissues. They exhibit distinct phenotypes compared to those in lymphoid tissues (18, 36).

## Tregs in skeletal muscle repair and regeneration

The role of Tregs in skeletal muscle repair and regeneration has become an increasingly important topic in immunology and regenerative biology. Originally known for their ability to suppress immune responses and maintain self-tolerance, Tregs are now seen as active players in tissue repair processes, including those in skeletal muscle (10, 11, 37, 38). Their regenerative functions have been documented in organs such as the skin (21, 39–41), lungs (42), heart (43–45), and intestines (46), and it is becoming clearer that Tregs also have a vital role in skeletal muscle regeneration after both acute and chronic injuries. Skeletal muscle has a notable capacity to regenerate following injury, mainly due to the presence of muscle stem cells, or satellite cells (MuSCs), located between the basal lamina and sarcolemma of muscle fibers (47–49). The process of muscle regeneration involves several precisely coordinated steps, including inflammation (50, 51), stem cell activation (52), differentiation (52, 53), and tissue remodeling (47). The immune system is crucial to this regenerative process, with immune cells not only helping clear debris and fight infection but also regulating MuSC activity through cytokines, growth factors, and direct cell interactions (53–55).

Following muscle injury, there is a rapid and temporary infiltration of various immune cells. Among these, a distinct subset of CD4^+^Foxp3^+^ Tregs accumulates at the injury site. Murine models of muscle damage, such as cardiotoxin-induced injury, show a significant rise in Treg frequency in the injured tissue within the first few days after injury (11). These muscle resident Tregs are phenotypically and functionally different from those found in lymphoid tissues, with unique transcriptional and epigenetic signatures compared to lymphoid organ Tregs. Single-cell RNA sequencing (scRNA-seq) studies have revealed that muscle Tregs form a distinct cluster enriched for genes involved in tissue repair, such as amphiregulin (AREG), Il1rl1 (encoding ST2), and Gata3, as well as increased expression of BATF and IRF4 transcription factors linked to tissue adaptation and repair (11, 56, 57). Muscle-resident Tregs are not only present in experimental injury models but have also been identified in chronic muscle disorders. In conditions like Duchenne muscular dystrophy (modeled by mdx mice) and myotonic dystrophy in humans, an accumulation of Tregs with similar gene expression patterns has been observed (56, 58). This supports the idea that skeletal muscle contains a reparative Treg population that reacts dynamically to injury and ongoing degeneration.

The role of Tregs in skeletal muscle regeneration is complex. One key function is controlling the innate immune response, especially the activity of neutrophils and macrophages. Neutrophils quickly respond to muscle injury and are crucial for removing dead tissue (38). However, they also release reactive oxygen species and proteases that can cause additional tissue damage (59). Tregs help regulate neutrophil activity and infiltration. In laboratory experiments, activated Tregs have been shown to boost the anti-inflammatory nature of neutrophils by encouraging the release of IL-10, TGF-β, haem oxygenase-1, and indoleamine 2,3-dioxygenase (9). These substances help resolve inflammation and create an environment that supports healing. The interaction between Tregs and macrophages is equally important. In skeletal muscle, macrophages go through a well-understood change in their behavior during regeneration. At first, they display a pro-inflammatory M1-like phenotype, producing TNF-α, IL-1β, and other cytokines that promote inflammation and activate MuSCs (60). As healing advances, macrophages switch to an anti-inflammatory M2-like phenotype, which secretes IL-10 and TGF-β to help tissue remodeling and reduce inflammation (60, 61). Tregs are crucial in encouraging this change by releasing IL-4, IL-10, and IL-13 (62, 63). This cytokine-driven shift not only dampens excessive inflammation but also supports the growth and development of MuSCs, directly aiding muscle repair.

Studies have also shown that Tregs help maintain the homeostasis of macrophage subsets in injured muscle. For example, Treg depletion causes a decrease in MHCII^+^ macrophages and an overexpansion of MHCII^+^ macrophages, which are linked to increased antigen presentation and interferon-γ (IFN-γ) responses (64). This imbalance boosts inflammation and disrupts the regenerative process. Conversely, Tregs help stabilize macrophage populations and reduce excessive IFN-γ signaling, thus creating a favorable environment for muscle regeneration (64). In addition to their role in modulating the innate immune response, Tregs also influence adaptive immunity during skeletal muscle repair. After injury, effector T cells, including Th1, Th2, Th17, and CD8^+^ T cells, infiltrate the damaged muscle and contribute to local immune responses (62). Tregs suppress excessive effector T cell activation through various mechanisms. One important pathway involves the downregulation of costimulatory molecules CD80 and CD86 on dendritic cells via contact-dependent interactions involving CTLA-4 and LAG-3 (62). This process induces indoleamine 2,3-dioxygenase in dendritic cells, which limits T cell proliferation and inflammatory cytokine production, ultimately supporting tissue repair (62).

**Figure 1:**
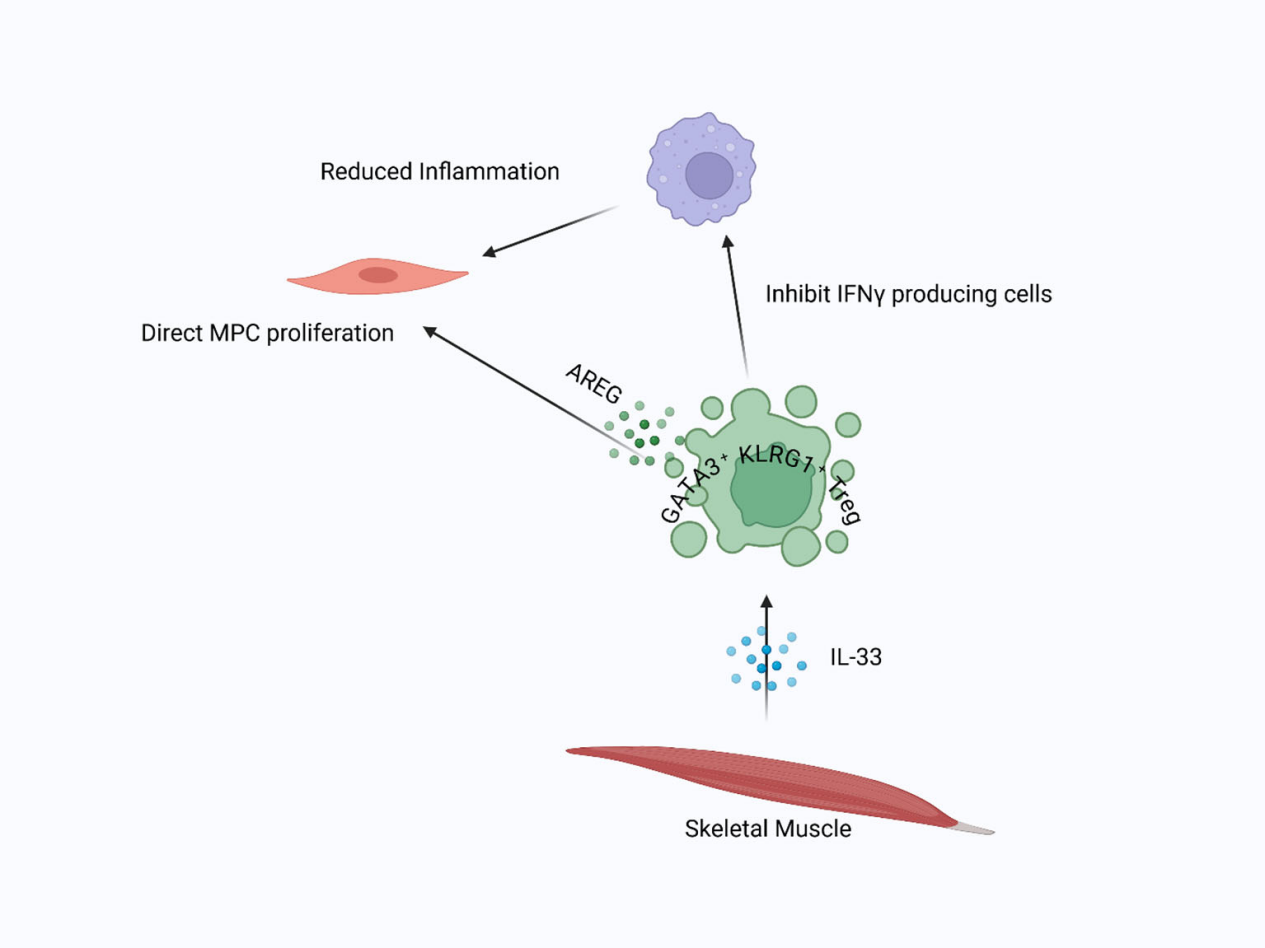
Skeletal muscle resident Tregs in injury repair: In injured skeletal muscle, IL-33 attracts Tregs to the damaged area. Once present, Tregs suppress inflammation driven by M1 macrophages and facilitate the transition to tissue repair. Additionally, Tregs directly stimulate satellite cell proliferation and differentiation by releasing AREG, supporting muscle regeneration.

Tregs also exert direct suppressive effects on effector T cells. They can downregulate IFN-γ production from Th1 and CD8^+^ T cells, which is crucial because IFN-γ drives macrophage activation toward a pro-inflammatory state by inducing CIITA and promoting MHCII expression (38, 43, 65–67). By limiting IFN-γ levels, Tregs help maintain a favorable balance of immune activation that encourages regeneration rather than chronic inflammation (64). This immunoregulatory loop involving Tregs, macrophages, and effector T cells is central to coordinating muscle repair. One of the most compelling mechanisms by which Tregs contribute to skeletal muscle regeneration is through the secretion of AREG, an EGFR ligand that acts directly on muscle satellite cells. AREG binds to the EGFR expressed on MuSCs, activating downstream signaling pathways that support satellite cell proliferation and survival (68). Burzyn et al. (2013) demonstrated that myogenic precursors exposed to AREG show enhanced regenerative potential (11, 69). This direct crosstalk between Tregs and muscle progenitor cells highlights a non-immune mechanism by which Tregs influence tissue regeneration (**Figure 1**). The importance of AREG has been supported in both injury and disease models. For example, mice subjected to cryoinjury show increased Treg numbers and elevated expression of AREG, IL-10, and TGF-β at the injury site, supporting the idea that Tregs promote tissue regeneration through a combination of immunomodulation and direct action on satellite cells (70). Similarly, in models of influenza-induced lung damage, Treg-specific deletion of AREG impairs tissue repair despite a normal antiviral immune response, indicating that the role of AREG is specifically regenerative (70). AREG treatment also improves muscle function and reduces Th1-driven inflammation, partly through effects on the transcription factor T-bet in Tregs and the promotion of CD206^hi^ Ly6C^low^ macrophages, further supporting its therapeutic potential (71).

Despite its regenerative benefits, AREG does not directly increase Treg proliferation or survival. Instead, it affects the post-translational regulation of Foxp3, leading to its degradation via EGFR–GSK-3β signaling pathways (72, 73). This feedback mechanism may serve as a regulatory checkpoint to prevent prolonged or excessive Treg activation. Additionally, AREG upregulates key myogenic markers such as Pax7, MyoD, and myogenin, which promote MuSC activation and differentiation during regeneration (71). Various external signals regulate Treg accumulation and activation in injured muscle. The IL-33–ST2 axis has been identified as a crucial regulator of Treg expansion at the injury site. IL-33, an alarmin released by stressed or damaged cells, enhances ST2 expression on Tregs, promoting their activation and retention (57, 74, 75). Mice lacking ST2 exhibit delayed Treg recruitment and impaired muscle regeneration, confirming the importance of this axis in tissue repair (57).

Furthermore, IL-33 may interact with neuro-immune pathways, as IL-33^+^ muscle mesenchymal stem cells have been shown to communicate with nerves and stromal elements, influencing Treg accumulation through CGRP signaling (76, 77). Importantly, IL-33 is not limited to Tregs; it can also affect other IL1RL1+ cells, such as type 2 innate lymphoid cells (ILC2s) (78), eosinophils, and alternatively activated macrophages (79), all of which assist in muscle regeneration. While Tregs are the primary responders to IL-33 during tissue repair, it remains crucial to study its diverse effects carefully for therapeutic purposes. Other regulatory molecules, such as PD-1 and sex hormones, also regulate Treg function in muscle. PD-1 is vital for the formation of peripherally induced Tregs, and its absence reduces Treg accumulation and delays repair (80, 81). Estrogen, on the other hand, promotes Treg recruitment and suppresses Th1-mediated inflammation, further supporting the reparative environment (82, 83). Whether reparative Tregs in skeletal muscle are thymus-derived or peripherally induced still needs investigation. However, studies using neuropilin-1 (Nrp1) expression as a surrogate marker suggest that most tissue-resident Tregs, including those in skeletal muscle, are thymus-derived (84). This distinction has therapeutic implications because thymus-derived Tregs may provide more stable lineage characteristics, while peripherally induced Tregs can potentially be generated *in vitro* from conventional CD4^+^ T cells for adoptive cell therapy.

## Role of Tregs in CNS repair and regeneration

Tregs are extensively studied for their vital role in maintaining immune balance and controlling inflammatory responses. Their role in tissue repair and regeneration has attracted significant interest, especially in the context of central nervous system (CNS) injuries. The CNS, known for its limited ability to regenerate, poses a unique challenge for repair processes. However, recent research highlights the potential of Tregs in supporting CNS repair and regeneration. In models of ischemic brain injury, Tregs have been found to infiltrate the CNS during the sub-acute to chronic phases, where they gather around the lesion site. Their presence is linked to better neurological outcomes, indicating a reparative role (85). One way Tregs may assist CNS repair is by modulating the inflammatory environment. Tregs release anti-inflammatory cytokines like IL-10 and TGF-β, which can inhibit the activity of pro-inflammatory cells such as microglia and astrocytes (86, 87). This inhibition is essential for minimizing secondary damage and creating a supportive environment for neural repair.

The specific mechanisms by which Tregs promote CNS repair are still being clarified, but several key processes have been identified. One important factor is the secretion of AREG, a molecule known for its tissue-protective and regenerative properties (35, 85). AREG produced by Tregs has been shown to reduce the activation of astrocytes, a type of glial cell that, when overly activated, can inhibit neural repair by forming a glial scar (85). By suppressing astrocyte activation, Tregs help prevent the formation of inhibitory scar tissue, thus supporting neuronal survival and axonal regrowth. In addition to AREG, Tregs in the CNS also express unique molecules that may contribute to their reparative functions. For instance, brain-infiltrating Tregs have been found to express high levels of serotonin receptor 7 (Htr7) (35), which enhances cAMP levels (88). cAMP is known to improve the proliferation of Tregs and boost their functional capabilities (89). Activation of Htr7 by serotonin or selective serotonin reuptake inhibitors (SSRIs) has been shown to increase Treg numbers in the brain, further supporting their role in CNS repair (35). This finding opens the door to potential therapeutic strategies that involve modulating Treg activity through pharmacological agents.

**Figure 2:**
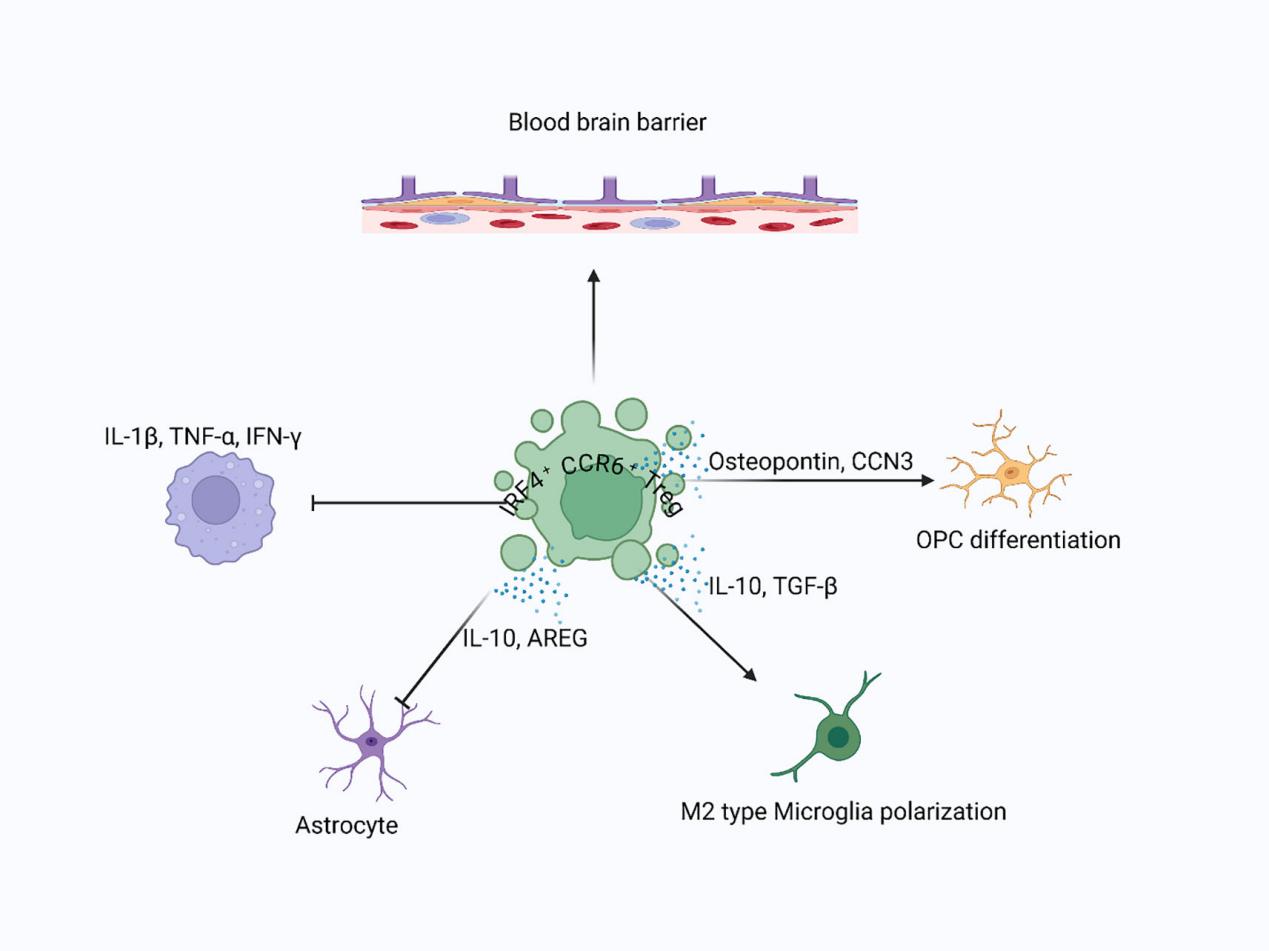
Treg-mediated recovery in CNS injury: Following CNS injury, Tregs infiltrate the brain parenchyma and interact with resident cells, including neurons, astrocytes, microglia, and oligodendrocyte progenitor cells (OPCs), to promote tissue recovery. Tregs suppress proinflammatory cytokines and stimulate the release of neuroprotective factors such as AREG, IL-10, and TGF-β, supporting remyelination, limiting glial scar formation, and enhancing debris clearance.

Tregs also interact with other CNS cells to promote repair. For instance, they influence the behavior of oligodendrocyte precursor cells (OPCs), which are responsible for remyelination. In experimental autoimmune encephalomyelitis models, transferring Tregs into Treg-deficient mice restores impaired remyelination (90), partly by secreting cell communication network factor 3 (CCN3), a protein that encourages OPC differentiation and myelination (90). This interaction between Tregs and OPCs highlights the diverse role of Tregs in CNS repair, extending beyond their traditional immune-regulatory functions. Another key interaction is between Tregs and resident CNS immune cells (macrophages, microglia, and γδT cells). Tregs can influence these macrophages (microglia), shifting them from a pro-inflammatory M1 phenotype to a more reparative M2 phenotype (35). This shift is crucial for clearing debris and supporting tissue regeneration after injury. Additionally, Tregs may also regulate the infiltration and activity of other immune cells, such as γδT cells and microglia, which participate in the inflammatory response after CNS injury (35). By modulating the immune response, aiding remyelination, and interacting with essential CNS cells, Tregs help create a supportive environment for tissue repair (**Figure 2**).

## Tregs in cardiomyocyte repair and regeneration

In lower vertebrates like zebrafish, Tregs are known to promote heart regeneration (91). However, in mammals, adult heart regeneration is limited due to the minimal proliferation of cardiomyocytes and is insufficient to repair large necrotic areas (92). Unlike the neonatal heart, which can regenerate during the first week of life—though the extent depends on injury size—this ability is absent in adults (93, 94). Despite this limitation, Tregs have been shown to contribute to heart repair in adults and support neonatal heart regeneration after myocardial injuries through various mechanisms (95, 96). For example, when Tregs are ablated using diphtheria toxin in FOXP3^DTR^ mice or depleted with an anti-CD25 antibody, adult mice experience significant heart deterioration after myocardial infarction (MI), including increased dilation of the left ventricles and impaired cardiac function (95, 96).

Furthermore, the absence of Tregs raises the risk of severe complications like apical aneurysm and cardiac rupture in adult mice post-MI (97). Similarly, depleting Tregs in neonatal mice results in increased fibrosis and reduced cardiomyocyte proliferation after injury, while adoptive transfer of Tregs promotes regeneration of neonatal heart cells by encouraging their proliferation (96). In adults, adoptive transfer of Tregs has been shown to improve cardiac function and reduce adverse remodeling after injury (96, 98). Additionally, activating Tregs through the administration of a super-agonistic anti-CD28 antibody (95). Collectively, these findings emphasize the essential role of Tregs in protecting the heart by minimizing adverse remodeling in adults and promoting regeneration in neonates.

Mechanistically, Tregs support heart tissue repair and improve cardiac function by regulating macrophage activity. *In vitro* studies have shown that Tregs encourage macrophages to adopt an M2-like phenotype (95), which is typically linked to tissue repair. However, this relationship depends on the context. In the neonatal heart, increased M2 macrophages have been seen in non-regenerating tissue after Treg depletion, while adoptive transfer of Tregs lowers M2 macrophages and enhances regeneration (96). Conversely, in the adult heart, Treg depletion reduces M2 markers and raises M1 cytokines after MI, indicating that M2 polarization benefits adult repair (95). These findings emphasize the dual role of M2 macrophages—pro-regenerative in adults but possibly pro-fibrotic in neonates—highlighting the need for a nuanced understanding of their function in cardiac recovery. This points to the importance of reconciling the pro-repair versus pro-fibrotic roles of M2 macrophages, especially when evaluating regenerative therapies across age groups.

In addition to modulating macrophages, Tregs can also suppress the pro-inflammatory responses of other immune cells, such as CD4^+^ and CD8^+^ T cells. Intriguingly, CD4^+^ T cells have a developmentally distinct role in heart repair and regeneration, where they inhibit heart regeneration in juvenile mice but support repair in adults (99). Unlike CD4^+^ T cells, CD8+ T cells are mostly unresponsive to heart injury in juvenile mice (99). Th_1_ and Th_17_ cells, known for producing pro-inflammatory cytokines like TNF-α, IFN-γ, and IL-17A, can directly inhibit cardiomyocyte proliferation and induce apoptosis in neonatal cardiomyocytes *in vitro*. Tregs likely contribute to heart regeneration by inhibiting these pro-inflammatory activities after myocardial injury.

**Figure 3:**
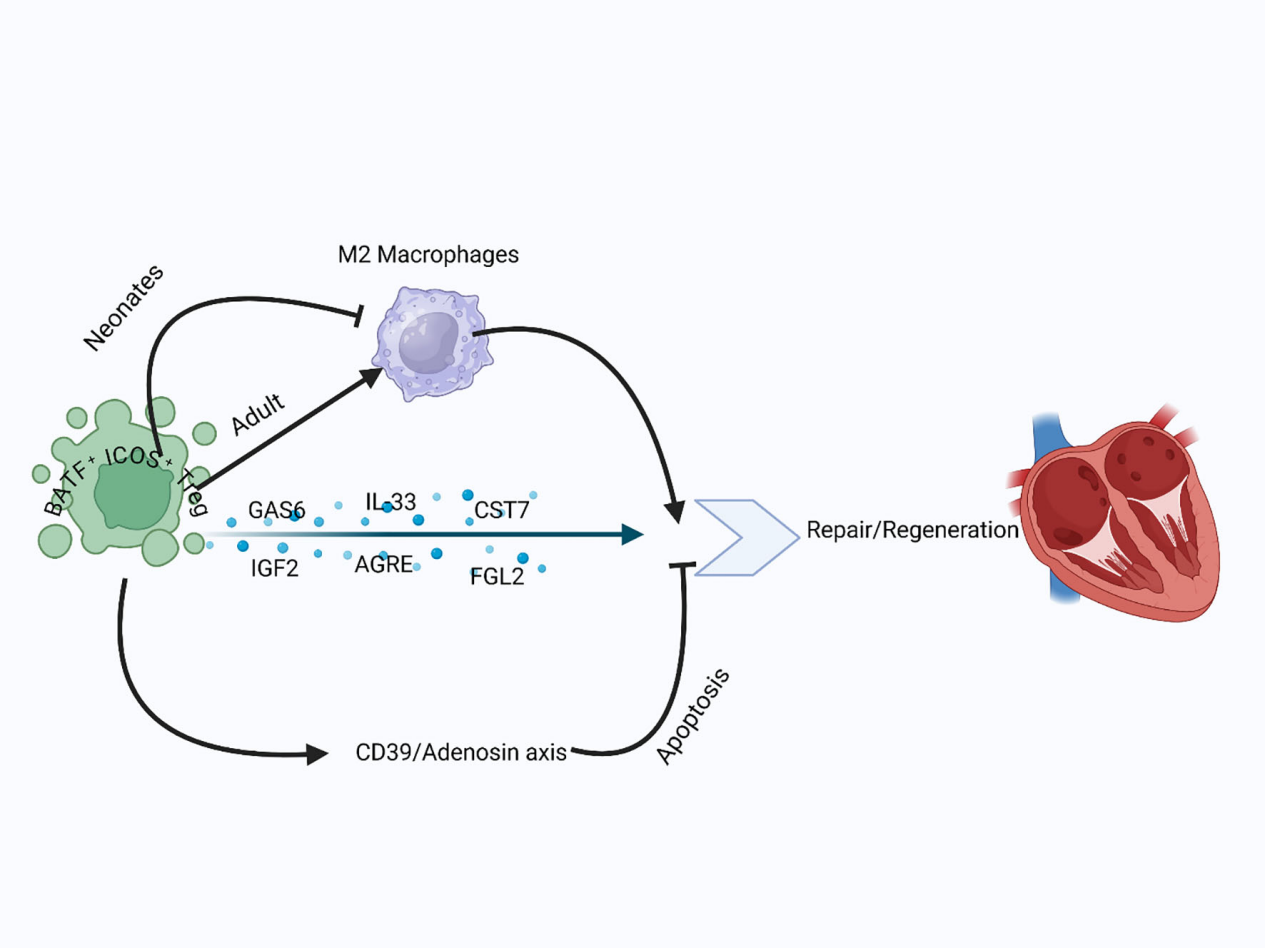
Treg role in cardiac repair and remodeling: In both neonatal and adult hearts, Tregs reduced adverse remodeling and enhanced cardiomyocyte proliferation. Tregs modulate M2 macrophage polarization, promoting repair in adults and limiting fibrosis in neonates. They reduce inflammation, inhibit apoptosis through the CD39–adenosine axis, and secrete paracrine factors like IGF2, CST7, and AREG to stimulate myocardial regeneration.

Tregs also help reduce cardiomyocyte apoptosis through a mechanism involving CD39. *In vivo* studies have shown that transferring CD39-deficient Tregs does not decrease infarct size after MI as effectively as transferring normal Tregs (100). CD39 is known to catalyze the production of adenosine, which replicates the protective effects of Tregs on cardiomyocytes (101). The adenosine produced via CD39/CD73 signaling activates the adenosine A_2A_ receptor, promoting mitochondrial stabilization, reducing oxidative stress, and preserving cellular ATP levels—effects critical for cardiomyocyte survival and function during ischemic stress (102). Furthermore, Tregs help prevent apoptosis of heart cells via the CD39/adenosine/reperfusion injury salvage kinase (RISK) pathway. The RISK pathway includes kinases such as Akt and ERK1/2, which collectively promote cardiomyocyte survival by preventing mitochondrial permeability transition pore opening and activating anti-apoptotic cascades (103). It is also important to distinguish between the reparative and anti-fibrotic functions of Tregs, as these represent distinct processes with separate molecular regulators. Reparative functions typically involve paracrine factors that support cardiomyocyte proliferation and survival (104). At the same time, anti-fibrotic effects stem from Treg-mediated suppression of fibroblast activation and ECM deposition through IL-10, AREG, and adenosine signaling (20, 105). Tregs play a crucial role in the regeneration of heart cells during the neonatal period by enhancing their proliferation. The limited ability of adult cardiomyocytes to regenerate can be linked to factors such as their binucleated state, which leads to cell cycle arrest, and the unique immune response observed in adults after injury. For instance, neonates exhibit a less dominant Th1 and Th17 response, instead favoring Th_2_-type immunity. The differentiation of Tregs from naive CD4^+^ T cells appears to be a natural default process in this context (106). Consequently, the impact of Treg-driven cardiomyocyte regeneration is more significant in neonates. The paracrine effects of Tregs seem to be a key mechanism, as the culture medium of Tregs alone can stimulate neonatal cardiomyocyte proliferation (96). Further analysis through single-cell RNA sequencing of Tregs after myocardial injury identified factors like CST7, MATN2, CCL24, GAS6, TNFSF11, FGL2, IGF2, AREG, IL33, as potential candidates that promote cardiomyocyte proliferation (96, 97). One important factor, AREG, secreted by Tregs, plays a key role in cardiac repair following myocardial infarction. Although it remains unclear whether AREG acts directly on cardiac progenitor cells, endothelial cells, or through intermediaries, its mitogenic and tissue-protective functions suggest broad regenerative roles. Treg-derived AREG has been shown to enhance cardiomyocyte proliferation and modulate macrophage polarization (96), and may also support endothelial cell function and neovascularization (104), highlighting its multifaceted contributions to heart regeneration. These findings expand the understanding of Tregs’ reparative abilities through their interactions with local cells like cardiomyocytes, enhancing tissue repair and regeneration. Overall, Tregs are crucial for both adult heart repair and neonatal heart regeneration, working through immune modulation, fibrosis reduction, prevention of apoptosis, and stimulation of cardiomyocyte proliferation (**Figure 3**).

## Functions and specialization of skin-resident Tregs

**Figure 4:**
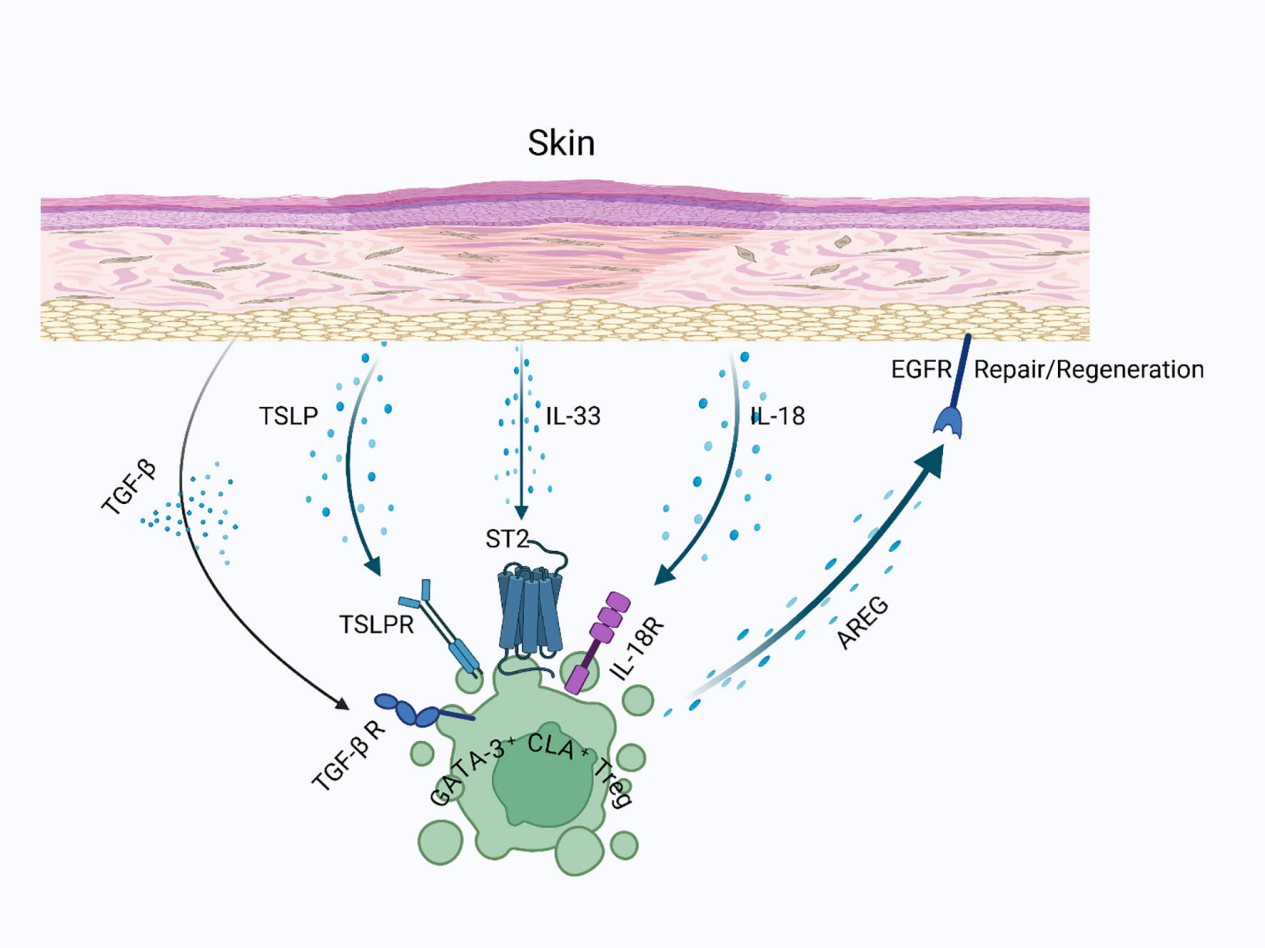
Skin-resident Tregs in wound healing: Skin-resident GATA-3-expressing Tregs express receptors for alarmins such as TSLP, IL-33, and potentially IL-18, which are released upon tissue injury. These signals enable Tregs to detect local damage and induce AREG production, promoting keratinocyte proliferation and facilitating the regeneration of stromal components in injured skin.

Skin-resident Tregs can differentiate into subsets similar to helper T cells (TH_1_, TH_2_, TH_17_) through specific transcription factors. Specifically, “Type 2” or “repair type” Tregs, marked by IRF4, GATA-3, and BATF, are essential for managing type 2 immune responses and supporting tissue repair (21, 107, 108). GATA-3 acts as a master regulator of type 2 immunity, while BATF (basic leucine zipper transcription factor ATF-like) further refines the reparative phenotype by promoting IL-10 and AREG production (109, 110). GATA-3^+^ Tregs not only suppress type 2 inflammation but also perform reparative functions associated with TH_2_ and ILC2 cells. These cells detect tissue damage via receptors for alarmins such as IL-33, IL-18, TSLP, and IL-25, which are released by stressed cells (111). In various tissues, including the skin (36), wounded muscle (11), and visceral fat (112), GATA-3^+^ Tregs express the IL-33 receptor ST2, enabling them to engage in repair mechanisms triggered by IL-33 from damaged cells, a process independent of TCR stimulation. ST2+ Tregs are more effective at promoting wound healing by regulating inflammation and enhancing epithelial regeneration (113). Similarly, TSLP, released by keratinocytes during inflammation, helps activate skin Tregs (114). IL-18, another key cytokine, might also influence skin Tregs’ reparative function, though the presence of its receptor on skin Tregs needs further confirmation (115, 116). Thus, skin-resident type 2 Tregs are specialized for utilizing type 2 immune mechanisms in tissue repair (**Figure 4**).

Skin injury triggers a strong inflammatory response that attracts immune cells, including Tregs, from the bloodstream to the damaged area. This response occurs not only in the skin but also in tissues that generally lack resident Tregs, such as the brain and muscle, emphasizing their role in healing (11, 85). Tregs gather in the skin after both minor and severe wounds, reaching their peak around one week post-injury (117, 118). The precise mechanisms behind Treg accumulation—whether through blood recruitment or local proliferation—are not entirely understood. Inflammation typically involves both processes and the movement of cells into the lymphatic system (119). Skin-resident Tregs respond promptly to injury, while circulating Tregs need time to activate and migrate, potentially supporting the repair process initiated by resident Tregs (21).

AREG, which interacts with epidermal growth factor receptor (EGFR), is essential for tissue repair and is produced by various type 2 immune cells, including GATA-3^+^ Tregs (120). The production of AREG in Tregs is stimulated by interleukins IL-18 and IL-33 (20). When bound to EGFR, AREG promotes the proliferation and differentiation of target cells, aiding in the repair and regeneration of damaged tissue. Interestingly, autocrine EGFR signaling in Tregs has been primarily described in skin wound healing (117), but emerging evidence suggests similar mechanisms operate in lung and muscle repair (11, 20). For example, in muscle injuries, AREG from Tregs supports the regeneration of myofibers from satellite cell progenitors (11). While several cell types, such as basophils, ILC2s, TH2 cells, and macrophages, can produce AREG, its expression by Tregs is particularly significant (20). Mice lacking AREG specifically in Tregs show increased mortality following influenza infection and extensive lung damage, highlighting the critical role of Tregs in AREG-mediated tissue repair (20).

Although research on Treg-derived AREG in skin injury is limited, evidence indicates its importance. Skin-resident Tregs express AREG (36), which promotes keratinocyte proliferation (120), similar to its effects on other epithelial tissues. AREG, along with keratinocyte growth factor (KGF) and fibroblast growth factor 2 (FGF2), synergistically enhances re-epithelialization by stimulating keratinocyte migration and proliferation (121). Additionally, these growth factors contribute to stromal remodeling by activating fibroblasts and promoting extracellular matrix reorganization (122). AREG also helps maintain vascular integrity by activating TGF-β signaling in pericytes (123), which may support stromal repair following skin injury. In wounded skin, Tregs express the EGFR receptor for AREG, and removing EGFR specifically from Tregs leads to fewer Tregs and slower wound healing, suggesting that AREG may have an autocrine effect in boosting Treg numbers during skin injury (117). Besides AREG, Tregs secrete other growth factors that aid repair by acting directly on non-hematopoietic cells. For example, Tregs release keratinocyte growth factor (KGF) to support alveolar regeneration after lung damage (124) and fibroblast growth factor 2 (FGF2) to enhance intestinal epithelial growth during colitis (125). In the skin, KGF and FGF2 likely contribute to epidermal repair by stimulating keratinocyte proliferation and migration. In zebrafish, Tregs help regenerate various organs after injury by secreting growth factors such as neuregulin 1 (NRG1) in the heart, nerve growth factor (NGF) in the spinal cord, and insulin-like growth factor 1 (IGF-1) in the retina (91). Both KGF and IGF-1 are also produced by dendritic epidermal T cells (DETCs) to support epidermal regeneration after skin injury (126). This suggests that skin-resident Tregs may employ similar mechanisms to facilitate repair processes following skin injury. The roles of IL-10 and TGF-β in skin repair seem to be both sequential and context-dependent. Early after injury, IL-10 predominates to suppress excessive inflammation, while TGF-β becomes more prominent in later stages to regulate fibrosis and promote matrix remodeling (127, 128). Tregs dynamically adjust their cytokine production in response to the wound microenvironment, ensuring a balanced healing response (117).

## Tregs in lung repair and regeneration

The lung’s ability to maintain homeostasis despite constant exposure to airborne pathogens and particulates reflects its complex immune-regulatory networks (129, 130). In disease states like infection-induced acute respiratory distress syndrome (ARDS), this balance is disrupted, requiring not only pathogen clearance and inflammation control but also regeneration of damaged tissue (131). This regenerative process involves carefully coordinated interactions among epithelial, endothelial, and mesenchymal compartments (132). Tregs, beyond their typical anti-inflammatory functions, gather in injured alveolar spaces in both mice and humans and actively aid in lung repair (133, 134). Among epithelial cells, alveolar type II (AT2) cells are crucial for regeneration because of their progenitor role—they proliferate, secrete surfactant, and differentiate into alveolar type I (AT1) cells to restore the gas exchange surface (130). Tregs support AT2 proliferation and survival through various mechanisms, including secreting keratinocyte growth factor (KGF) and engaging in direct cell interactions. Specifically, Mock et al. (2014) showed that Tregs expressing CD103 (αE integrin) physically interact with AT2 cells via E-cadherin, a key adhesion molecule on epithelial cells (135). This CD103/E-cadherin axis helps retain Tregs within the alveolar niche and enables localized communication with AT2 cells. Co-culture experiments further support this interaction. *In vitro* studies using isolated Tregs and primary AT2 cells show that CD103^+^ Tregs promote AT2 proliferation and maintain their stem-like transcriptional profile, partly through E-cadherin-dependent adhesion (135, 136). When CD103 or E-cadherin is disrupted in these systems, Tregs’ ability to promote epithelial regeneration decreases, indicating that this interaction is not just structural but also essential for Treg-driven epithelial repair. Importantly, this adhesion-dependent mechanism likely allows close-range delivery of trophic signals, such as KGF and AREG, enhancing repair pathways in a context-specific manner (124, 135).

AREG, an EGFR ligand, has become an important mediator of Treg-driven tissue protection and repair in the lungs and other tissues (11, 20, 137, 138). In a mouse model of influenza-induced lung injury, IL-18 and IL-33 signaling promote Treg-derived AREG production, which helps preserve alveolar structure and gas exchange (136). Interestingly, the IL-18/IL-33-induced AREG expression seen in lung Tregs mirrors similar mechanisms in skin and gut tissue-resident Tregs. In the skin, AREG production by GATA3+ Tregs promotes keratinocyte proliferation, while in the gut, AREG supports maintaining the epithelial barrier during colitis (20). However, the lung microenvironment, rich with alveolar macrophages and surfactant-secreting epithelial cells, may uniquely boost IL-18/IL-33 signaling, making lung-resident Tregs especially powerful producers of AREG after infection or injury (68). This tissue-specific response could explain the strong repair response observed in the lungs. NOTCH4 is a key transcriptional regulator of IL-18–induced AREG expression in Tregs, and its levels are inversely related to serum AREG levels and COVID-19 severity, linking this pathway to clinical outcomes (138). Furthermore, a mesenchymal cell population expressing Col14a1 has been identified as a target of Treg-derived AREG signals, supporting alveolar epithelial organoid growth and aiding tissue repair *in vivo* (139). Deletion of EGFR in these stromal cells worsens hypoxia after influenza infection, indicating that Treg-derived AREG acts on mesenchymal targets as well.

However, mesenchymal cell populations are heterogeneous. While some subsets support epithelial regeneration, others (e.g., AXIN2^+^ myogenic precursors) can worsen injury by promoting fibrosis through myofibroblast activation (140). Tregs help control this maladaptive response. During the fibroproliferative phase of acute lung injury, Tregs decrease the recruitment of collagen-producing bone marrow–derived cells by modulating CXCL12/CXCR4 signaling (141). Pharmacologic inhibition of CXCR4 with AMD3100 reduces fibrosis independently of CXCL12, indicating CXCR4 as a potential therapeutic target. While epithelial repair has been a primary focus, endothelial regeneration is equally important for restoring lung homeostasis. Coordinated signaling between epithelium and endothelium is crucial for regenerating the alveolar-capillary interface (142). CAR4^+^ endothelial cells, which depend on VEGFA signaling from epithelial cells, play a vital role in this process during both development and post-viral injury (143, 144). These cells are enriched in tip-cell markers (Vegfr2, Nrp1, Apln) and are located near AT1 cells, suggesting their involvement in vascular regeneration.

**Figure 5:**
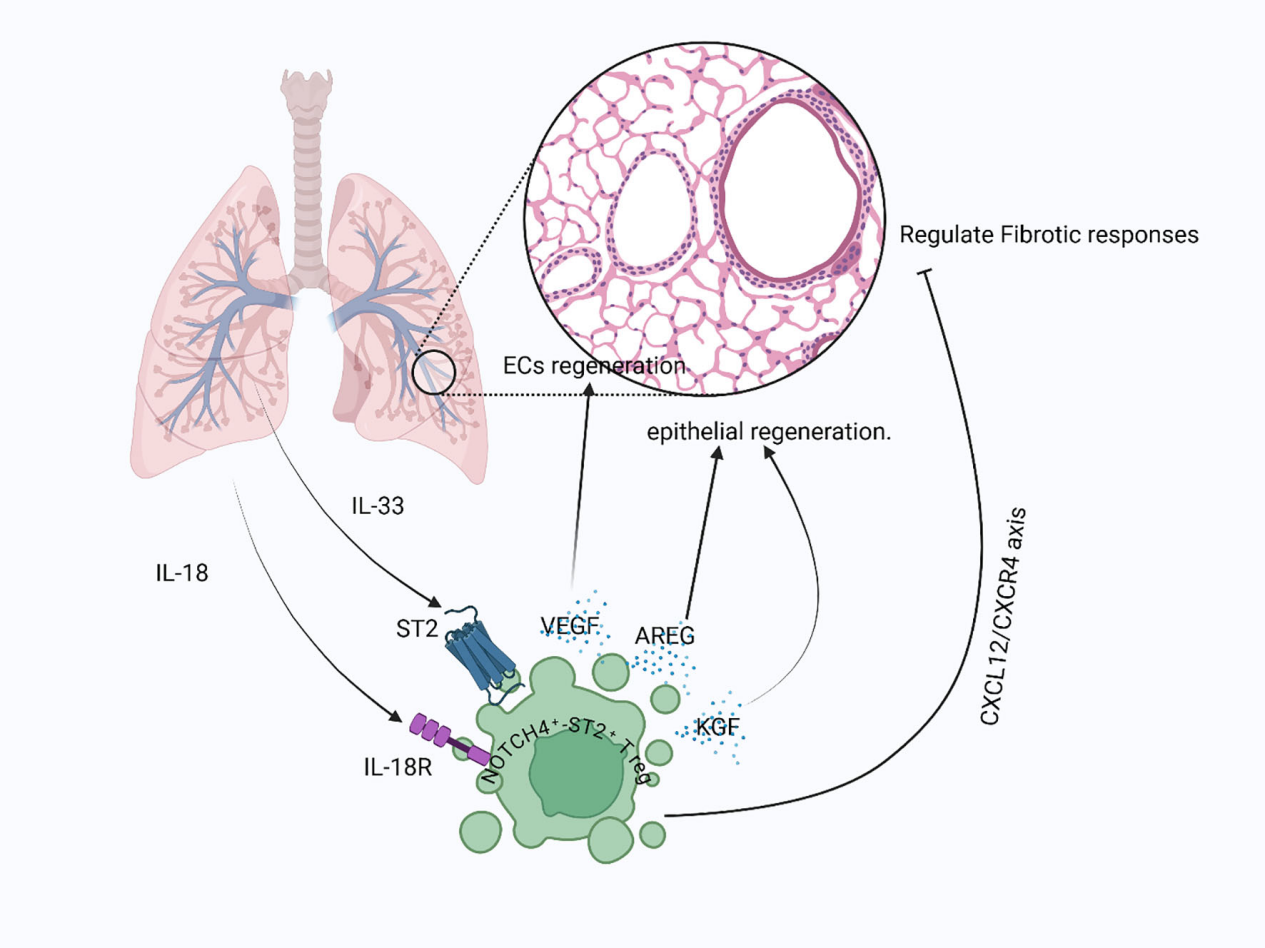
Treg-mediated lung repair post-injury: After lung injury, signals like IL-18 and IL-33 released from damaged epithelial cells and activated immune cells interact with their respective receptors on Tregs, stimulating the production of epithelial-repair-promoting factors such as AREG and KGF. Tregs also contribute to vascular repair by releasing VEGF to support alveolar capillary endothelial cell recovery, while limiting fibrotic responses through the CXCL12/CXCR4 signaling axis.

Beyond acute injury, Tregs also have roles in chronic lung diseases. In pulmonary fibrosis models, Tregs regulate fibroblast activity and reduce excessive collagen buildup through TGF-β-dependent and independent pathways (145). In chronic obstructive pulmonary disease (COPD), lower Treg numbers and impaired function are linked to ongoing inflammation and alveolar damage (146). These findings indicate that improving or restoring Treg function could be important for preventing or treating chronic lung remodeling and dysfunction. Tregs have multiple roles in lung repair, including secreting trophic factors (KGF, AREG), directly interacting with epithelial cells via CD103/E-cadherin, controlling fibrotic responses through the CXCL12/CXCR4 axis, and supporting endothelial regeneration (**Figure 5**).

## Tregs in bone repair and regeneration

**Figure 6:**
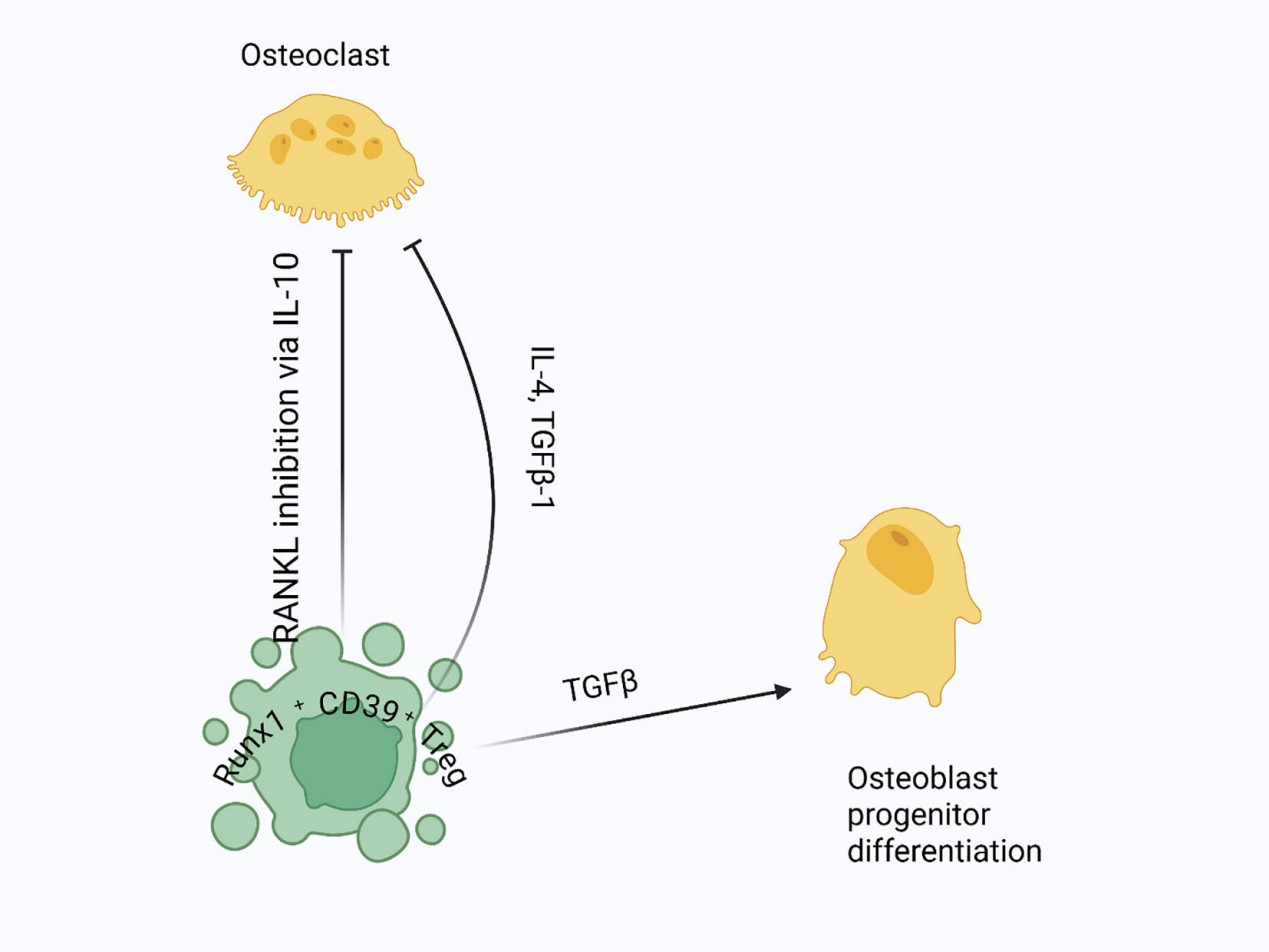
Treg regulation of osteoclastogenesis and bone formation: Tregs suppress osteoclastogenesis by inhibiting RANKL signaling, crucial for osteoclast differentiation and activation. They secrete anti-inflammatory cytokines like IL-10 to block osteoclast precursor maturation, and they release TGF-β and IL-4 to further inhibit osteoclast differentiation. Additionally, Tregs produce TGF-β to support osteoblast differentiation and bone formation.

Tregs have proven to be key players in bone healing and regeneration. Unlike many tissues that heal with scar tissue, bone has a remarkable ability to regenerate without scarring through the combined action of osteoblasts and osteoclasts (9). Osteoblasts, originating from mesenchymal stem cells (MSCs), build new bone, while osteoclasts, derived from bone marrow cells, break down old bone. Tregs help regulate this process by influencing both bone formation and breakdown [131]. Mechanistically, Tregs suppress osteoclastogenesis mainly by inhibiting receptor activator of nuclear factor kappa-B ligand (RANKL) signaling, which is crucial for osteoclast differentiation and activation. This suppression occurs via secretion of anti-inflammatory cytokines like IL-10, which inhibit osteoclast precursor maturation and function (147, 148). Tregs also produce TGF-β, contributing to osteoblast differentiation and supporting bone formation (149). Although direct interactions of Tregs with osteoblast precursors are less well defined, recent evidence suggests Tregs may promote osteoblast maturation indirectly by modulating the inflammatory environment to favor bone growth (150). MSCs can induce Tregs to develop from naive T-cells and help them multiply, partly through a protein called haeme oxygenase-1 (HO-1) (151). Notably, this MSC-induced Treg differentiation appears to be largely antigen-independent, relying instead on paracrine signals like TGF-β and prostaglandin E2, which create an immunosuppressive environment conducive to Treg expansion (152). Moreover, CD3^+^ T-cells aid in the differentiation of other blood cells into osteoclasts, and Tregs intervene to inhibit this process, using signaling molecules such as TGF-β and IL-4 (148, 153) (**Figure 6**). This inhibition is important because higher levels of Tregs in the blood are associated with lower levels of markers indicating bone loss, a trend observed in both healthy individuals and those with rheumatoid arthritis. Animal studies also support the idea that Tregs help prevent bone destruction. For instance, Tregs can protect against bone loss caused by TNF-α and reduce bone damage in ovariectomized mice (154, 155). Using specific antibodies to enhance Treg activity has also shown promise in reducing arthritis and increasing bone density in mice, likely by limiting osteoclast activity (9, 156).

Tregs may also directly aid in the maturation of bone-forming cells. In a bacterial-induced bone injury model, Tregs were drawn to the injury site by a signaling molecule called CCL22. There, they helped prevent bone loss by reducing inflammation (156). Additionally, Tregs support MSC-based bone repair by suppressing CD4^+^ T-cells that produce inflammatory cytokines like IFN-γ and TNF-α, which can impede bone formation (157, 158). Therefore, Tregs are vital for bone repair and regeneration. They not only help regulate bone resorption but also promote new bone growth, making them essential for maintaining bone health and facilitating healing.

## Role of Tregs in liver diseases

**Figure 7:**
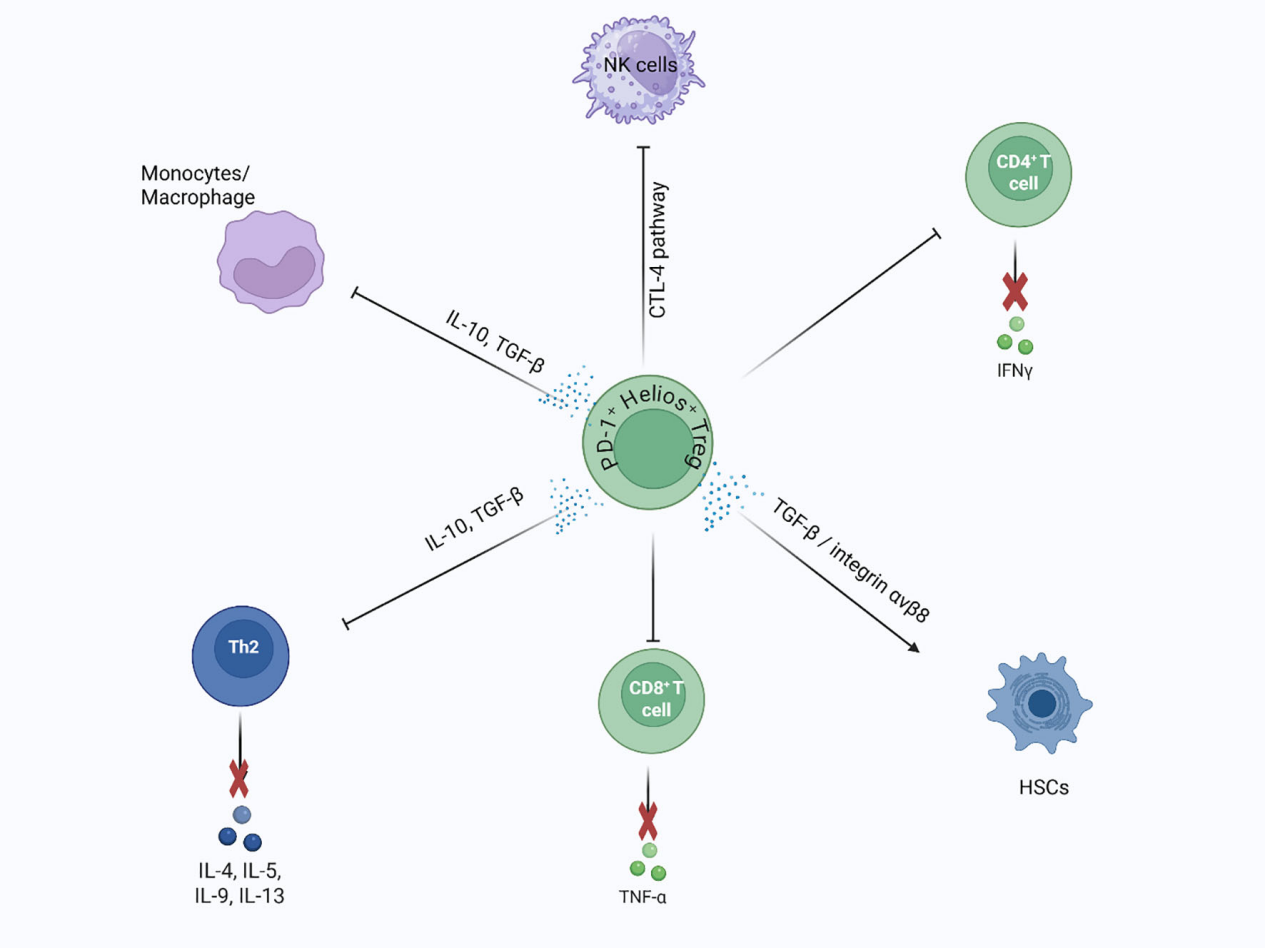
Dual role of liver Tregs in fibrosis regulation and repair: Liver Tregs (Helios^+^, ST2^+^) play a dual role in liver fibrosis and regeneration. During repair, hepatic stellate cells (HSCs) promote Treg regeneration via MMP 9/13-dependent TGF-β activation, supporting wound healing. In contrast, liver Tregs suppress pro-fibrotic immune cells, such as Th2 and Ly-6Chigh monocytes/macrophages, through IL-10 and TGF-β secretion. They also inhibit CD8^+^ T cell proliferation and modulate HSC activity by suppressing NK cells, influencing fibrosis progression.

When chronic wounds persist and cause ongoing inflammation, they can trigger a series of events that lead to liver fibrosis and eventually cirrhosis (159). Different liver inflammatory diseases, such as non-alcoholic fatty liver disease (NAFLD), non-alcoholic steatohepatitis (NASH), hepatitis B (HBV), and hepatitis C (HCV), contribute to liver fibrosis (160, 161). Reduced activity of Treg cells (indicated by low FoxP3 mRNA levels) has been observed in the NAFLD mouse model of the liver. This decline negatively affects NAFLD progression by disrupting the balance between Tregs and Th17 cells, resulting in an increased Th17-driven inflammatory response (162). An association has also been noted between an increased number of Foxp3^+^ lymphocytes in liver lobules and NAFLD-driven hepatic inflammation, further indicating the role of Tregs in hepatic disease (163). Similarly, fewer Tregs was observed in mice with steatohepatitis, but adoptively transferring Tregs into these mice helped reduce liver injury and slow disease progression (164). In autoimmune liver diseases, the number of infiltrating Tregs in the liver surpasses that circulating in the bloodstream (165). In these conditions, persistent inflammation causes hepatocyte apoptosis and necrosis. Consequently, the anti-inflammatory potential of Tregs helps mitigate this damage, providing a protective effect against liver inflammation (166). Studies also suggest that Tregs can contribute to liver fibrosis by influencing Kupffer cells through the TGF-β pathway (167, 168). Treg-derived TGF-β appears to act mainly through an indirect mechanism — modulating Kupffer cell polarization rather than acting directly — thereby shaping the immune microenvironment toward a more fibrotic or anti-fibrotic state (167, 169–171). Recent research proposes a dual role for Tregs in liver fibrosis: initially offering anti-inflammatory effects that protect hepatocytes, but later contributing to fibrotic progression (172, 173). In early NAFLD/NASH, Tregs suppress Th17-driven inflammation and inhibit hepatic stellate cell (HSC) activation (172, 173). However, in advanced fibrosis, they may switch roles (**Figure 7**). For example, AREG-expressing Tregs have been shown to directly stimulate EGFR signaling in HSCs, promoting fibrosis in both human and mouse models of NASH (171). This underscores a stage- and context-dependent shift from protective to pathogenic Treg activity. Single-cell RNA sequencing from mouse models and spatial transcriptomics from human liver tissue both reveal the presence of Tregs with a profibrotic gene signature, such as Areg and CTLA-4, which closely interact with hepatic stellate cells during fibrosis. This supports the idea that Tregs in fibrotic livers not only suppress inflammation but also actively contribute to fibrosis through cellular interactions and signaling (174, 175).

Tregs play a vital role in wound healing and reducing inflammation. However, some studies indicate that lowering Tregs could help reverse fibrosis. Activated HSCs release IL-2, which increases Tregs in fibrotic tissue. In a healthy liver, Kupffer cells (KCs) secrete matrix metalloproteinases (MMPs) that break down and regulate the liver’s ECM. Tregs produce TGF-β, which can inhibit KCs-mediated MMP release, potentially hindering fibrosis reversal (172). Research using DEREG transgenic mice, which lack Tregs, showed that removing these cells led to increased liver fibrosis, mainly due to an increase in CD8^+^ and IL-17A^+^ T cells and higher secretion of inflammatory factors (161). TGF-β is an essential cytokine that influences whether T cells develop into Th17 cells or Tregs. When TGF-β is low along with IL-6, it promotes Th17 cell differentiation. Conversely, high levels of TGF-β cause naïve CD4+ T cells to develop into Tregs. During liver fibrosis, both IL-6 and TGF-β levels increase, stimulating HSCs to produce more ECM proteins. This process heightens Th17 cell numbers and creates an imbalance between Tregs and Th17 cells (160).

## Tregs in kidney repair and regeneration

Tregs, known for their immune-modulatory functions, have increasingly been recognized for their key role in renal injury repair and regeneration, especially after ischemia-reperfusion injury (IRI). Studies show that Tregs infiltrate the kidneys during the healing process and are crucial in reducing damage and aiding recovery. In a murine model of ischemic acute kidney injury, Tregs were observed to migrate into the kidneys within 3 to 10 days following the injury. Their presence was linked to less renal tubular damage, increased tubular proliferation, and lower production of pro-inflammatory cytokines (176), all of which contributed to better renal function and lower mortality. Tregs primarily protect by suppressing excessive immune responses that can worsen renal injury. For example, depleting Tregs in animal models before inducing renal IRI resulted in more kidney damage, with higher blood urea nitrogen (BUN) and serum creatinine (Scr) levels, along with increased tubular necrosis scores. Conversely, infusing Tregs after IRI onset significantly enhanced renal repair by decreasing inflammatory cytokine production and supporting the recovery of renal function (177).

In addition to their immunosuppressive functions, Tregs also support tissue regeneration. In models of renal fibrosis, Tregs induced by mesenchymal stem cells (MSCs) pretreated with interferon-gamma (IFN-γ) were found to significantly decrease fibrosis and improve kidney function. This effect was mediated by the enzyme indoleamine 2,3-dioxygenase (IDO), whose expression was increased by IFN-γ (178). IDO played a key role in the anti-fibrotic effects of Tregs, indicating a potential therapeutic approach for preventing renal fibrosis. Further evidence emphasizes the role of Tregs in chronic kidney disease and transplantation. Foxp3^+^ Tregs have been shown to limit autoimmune kidney disease and aid in transplant tolerance. In kidney transplant models, depleting Tregs disrupted transplant tolerance, while their presence was linked to higher levels of immunosuppressive cytokines like TGF-β and IL-10 (179). This indicates that Tregs are not only vital for repairing acute injury but also crucial for maintaining long-term kidney health. It is important to distinguish the role of Tregs in acute versus chronic kidney conditions. In acute settings such as IRI, Tregs mainly work to suppress inflammation and protect renal tubular epithelial cells, primarily within the peritubular interstitium, thus promoting regeneration (180, 181). In contrast, in chronic conditions like renal fibrosis or transplantation, Tregs display functional plasticity and phenotypic diversity, driven by the inflammatory and alloimmune microenvironment. These cells may show altered FOXP3 stability, demethylation patterns, or cytokine expression—shifting between protective immune regulation, promotion of fibrotic processes, or tolerance induction after transplantation (181–183).

The mechanisms by which Tregs contribute to renal repair involve several pathways. One key process is suppressing innate immune responses, especially those mediated by neutrophils and macrophages (180), which are often elevated after IRI and contribute to ongoing tissue damage. Tregs accomplish this by secreting anti-inflammatory cytokines such as IL-10 and producing adenosine via CD73, which activates the adenosine A_2A_ receptor (A_2A_R) on immune cells to further reduce inflammation. The CD73/adenosine A_2A_ receptor (A_2A_R) axis plays a crucial role in controlling immune cell infiltration and supporting tubular epithelial cell survival. During injury, Tregs express CD73, converting extracellular AMP into adenosine. The adenosine then binds to A_2A_R on neutrophils and macrophages in the peritubular interstitium, decreasing their recruitment and cytokine production, thus creating a microenvironment (184).

**Figure 8:**
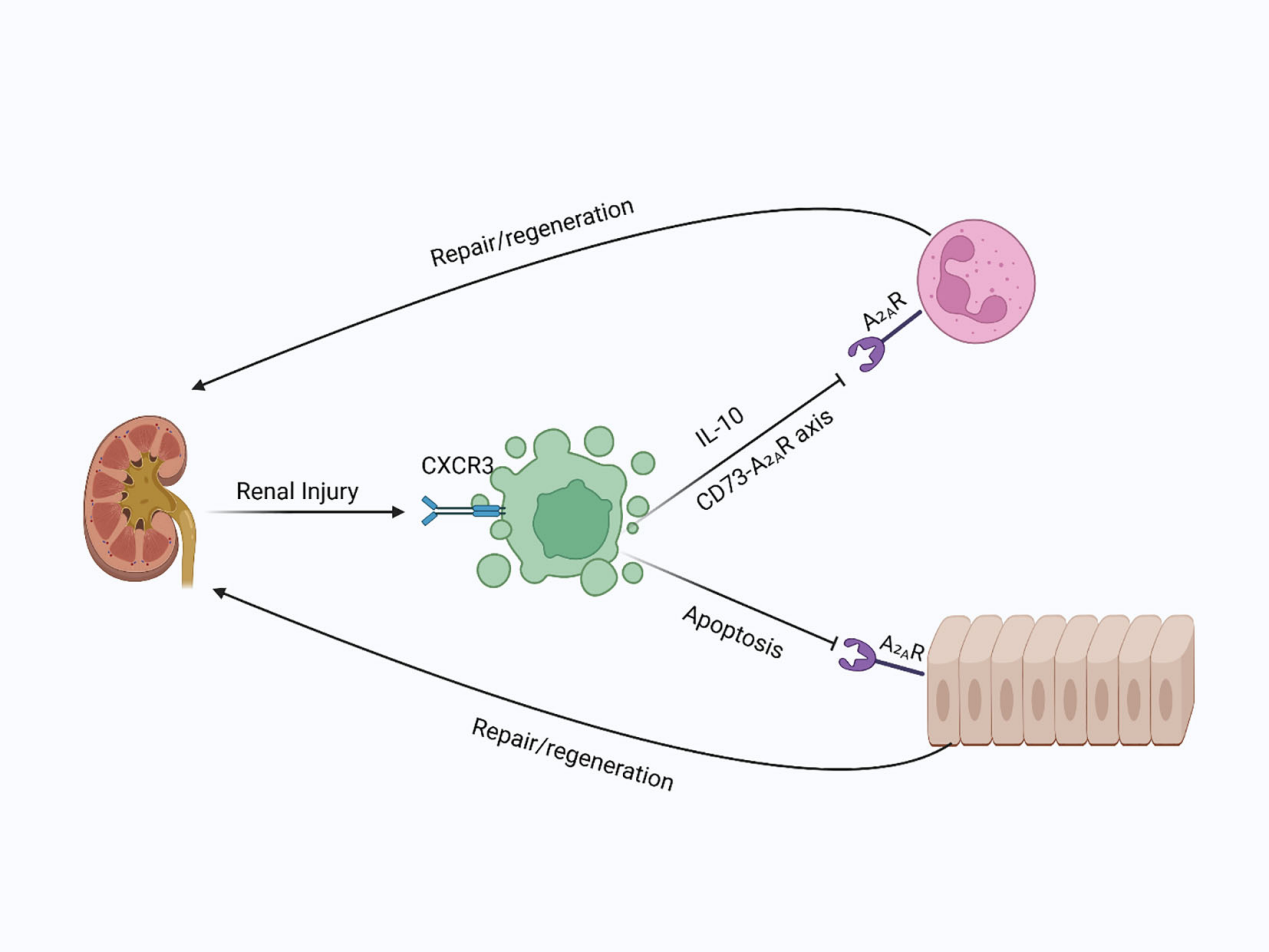
Tregs in kidney injury and repair: In kidney injury, Tregs are recruited to the site of damage, where they suppress immune responses and promote repair. By secreting IL-10 and producing adenosine via CD73, Tregs activate A2A receptors on immune and epithelial cells, inhibiting immune infiltration and apoptosis, leading to enhanced kidney regeneration.

Furthermore, activation of A_2_AR on renal epithelial cells and immune cells enhances tubular epithelial cell resistance to apoptosis and encourages proliferation, aiding kidney repair (185). Animal studies demonstrate the significance of this pathway: in models of ischemic kidney injury, Tregs lacking CD73 or A_2_AR fail to provide protection, whereas pharmacological activation of A_2A_R restores their protective function (184). Additionally, recent research emphasizes the role of chemokine receptors like CXCR3 in recruiting and functioning of Tregs during renal injury. CXCR3 expression on Tregs is linked to their migration into the kidney and subsequent improvement of IRI. Notably, CXCR3 is mainly found on activated effector Tregs, which helps them home to inflamed renal tissues. Evidence from human T-cell studies suggests that a small subset of CXCR3^+^ naïve-like T cells, which have ready effector potential, may upregulate CXCR3 after injury—indicating that even naïve or central-memory Tregs can gain homing ability during inflammation (186, 187). Mice overexpressing CXCR3 showed increased Treg infiltration, reduced inflammation, and better renal function (177, 186), highlighting the importance of chemokine signaling in Treg-mediated protection during renal IRI. Therefore, Tregs are essential for renal injury repair and regeneration by modulating immune responses, reducing inflammation, and promoting tissue regeneration. Their therapeutic potential is evident in both acute and chronic kidney diseases, making them a promising target for future therapies aimed at enhancing kidney repair and preventing fibrosis (**Figure 8**).

## Role of Treg in the gastrointestinal tract

Tregs are increasingly recognized not only for their immunosuppressive abilities but also for their active role in gastrointestinal (GI) tissue repair and regeneration (188). Instead of passively maintaining tolerance, Tregs dynamically adjust to environmental cues in the inflamed gut, where they play key roles in managing damage, preserving epithelial integrity, and supporting tissue renewal (46). During mucosal injury or infection, Tregs are recruited to affected sites, where they help control excessive immune activation. By secreting anti-inflammatory cytokines like IL-10 and TGF-β, they reduce collateral tissue damage by modulating both innate and adaptive immune responses (189, 190). Importantly, their functions go beyond immune suppression. In the intestinal mucosa, Tregs support the survival of intestinal epithelial cells (IECs) (22), promoting barrier stability and preventing microbial translocation. These effects are partly mediated through IL-10-dependent pathways that strengthen tight junctions and prevent IEC apoptosis (191, 192).

In response to inflammatory signals and epithelial-derived alarmins such as IL-33, a subset of Tregs expressing the transcription factor GATA3 becomes functionally active. These GATA3^+^ IL-33R^+^ Tregs are essential for reducing immunopathology and aiding tissue recovery during infection and inflammation (78, 193). Their accumulation is vital for maintaining mucosal balance, especially in the small intestine, where losing GATA3 expression impairs Treg localization and function during enteric infections (193). These cells show transcriptional stability even in germ-free conditions, indicating a thymic origin and a specialized adaptation to the GI environment (194, 195). Tregs also reprogram the inflammatory microenvironment to support regeneration. One key process involves shifting macrophage phenotypes from pro-inflammatory M1 states to reparative M2 profiles (196, 197). This shift improves debris clearance and fosters conditions favorable for tissue remodeling (112). Additionally, Tregs produce several paracrine mediators, including AREG (198, 199), insulin-like growth factor 2 (IGF-2) (200), and CST7, all of which aid epithelial repair and functional recovery (188). The environment in which Tregs operate is crucial. While they protect against inflammation-related damage during infection or injury, their presence in the tumor microenvironment, such as in colorectal cancer (CRC), can have different effects. Depending on the cytokine environment and subset type, Tregs may either suppress tumor-promoting inflammation or facilitate immune evasion by reducing anti-tumor immunity (201, 202). Collectively, Tregs are increasingly recognized as critical mediators of gastrointestinal tissue regeneration, functioning at the interface between immune regulation and epithelial restoration.

## Role of Treg in oral mucosa

The oral mucosa represents a unique immunological niche, continuously exposed to dietary antigens, commensal microbes, and mechanical stress. Within this dynamic environment, Tregs play a crucial role in maintaining immune balance and preserving epithelial integrity (203). Unlike other mucosal sites where local Treg induction is prominent, the oral mucosa is mainly populated by circulating Tregs that are recruited and acquire tissue-specific phenotypes, notably expressing CD103 and high levels of CTLA-4 to support retention and function within the oral tissue microenvironment (203). Mechanistically, Tregs suppress local effector T cell functions (Th1 and Th17). In Foxp3 knockout or IL-2 knockout mice (both of which lack functional Tregs), researchers observed a significant increase in Th1 (IFNγ^+^) and Th17 (IL-17A^+^) cells in the cervical draining lymph nodes, along with histological evidence of severe mucosal inflammation and damage, compared to wild-type controls (204). These findings emphasize that Tregs actively limit pathogenic T cell expansion to prevent mucosal injury.

Furthermore, the oral microenvironment is unique in that it minimally induces local peripheral Treg development; instead, the mucosa is mainly populated by recruited peripheral Tregs that adopt a tissue-resident phenotype (CD103^^+^,^ CTLA-4 ^high^) *in situ*, thereby maintaining homeostasis despite continuous exposure to food antigens, commensals, and mechanical stress (203). Tregs also influence microbiome-driven mucosal immunopathology. In murine models of oropharyngeal candidiasis, antibiotic disruption of resident bacteria resulted in decreased Foxp3^+^ Tregs and IL-17A^+^ cells, leading to worsened oral mucosal pathology (205, 206). Supplementing with short-chain fatty acids restored Treg frequencies (including Foxp3^+^IL-17A^+^ “Treg17” cells) and reduced tissue damage—showing that microbiota-derived metabolites support Treg-mediated protection of oral barrier tissues (206). The balance between Treg suppression and Th17 immunity is especially important. Some evidence indicates Tregs in the oral mucosa can promote protective Th17 responses by consuming IL-2 (a negative regulator of Th17 differentiation) and by producing TGF-β, thus allowing controlled IL-17 production that supports barrier immunity without causing pathological inflammation (207). This suggests that Tregs manage a finely tuned balance between tolerance and defense.

In disease contexts such as oral lichen planus (OLP), the role of Tregs appears complex. Immunohistochemical analysis of FOXP3^+^ Tregs in OLP lesions revealed correlations with disease activity, suggesting that Tregs may be either insufficient or dysregulated in chronic inflammatory conditions of the oral mucosa (208). Functional studies further support this, showing that FOXP3^+^ Tregs in OLP patients express reduced levels of immunosuppressive cytokines like TGF-β and demonstrate impaired suppressive capacity, indicating a loss of regulatory efficiency despite elevated cell counts (209). While CD8^+^ cytotoxic T cells mediate keratinocyte apoptosis in OLP, inadequate Treg suppression likely contributes to unchecked local immune activation. In contrast, evidence from celiac disease further illustrates the dynamic role of Tregs in oral mucosal injury. Sanchez-Solares et al. (2021) demonstrated that celiac disease disrupts oral epithelial integrity, shown by reduced E-cadherin and claudin-1 expression. This epithelial injury was accompanied by an increased infiltration of Foxp3^+^ Tregs and elevated expression of AREG, a key tissue repair molecule. The positive correlation between Treg presence and Areg expression suggests a compensatory, pro-repair function of Tregs in response to mucosal damage (210). Together, these findings indicate that Tregs may play dual roles in oral mucosal diseases—being insufficient or dysfunctional in chronic inflammation like OLP, while adopting a reparative phenotype in barrier-restoration settings such as celiac disease.

## Role of Treg in genitourinary

The genitourinary (GU) tract, including urinary and reproductive organs, requires a finely tuned immune balance to tolerate beneficial agents like sperm and commensals while defending against pathogens such as sexually transmitted infections. Tregs have emerged as central mediators in orchestrating this immune balance, modulating immune responses to favor tissue protection and homeostasis within the GU tract (46). For a successful and healthy pregnancy, the maternal immune system must accept both sperm and the fetus, which expresses paternal antigens and is therefore partially foreign. Tregs present in maternal blood and at the maternal–fetal interface has been shown to expand during gestation, playing a crucial role in preventing fetal rejection, spontaneous abortion, and complications like preeclampsia in both humans and animal models (211–214). In mice, Treg accumulation begins shortly after first contact with seminal antigens, even before embryo implantation, with recruitment seen in the uterus and draining lymph nodes (215–217). During pregnancy, the number of CD25^+^ T cells doubles in iliac and inguinal lymph nodes, and Tregs make up roughly 30% of uterine CD4^+^ T cells. Additionally, uterine Foxp3 expression increases nearly 1,000-fold compared to non-pregnant counterparts, indicating a significant surge in local Treg populations (211). Treg deficiency early in gestation has been linked to impaired uterine artery remodeling, supporting their role in preventing hypertensive disorders like preeclampsia (218). Immune suppression observed during the luteal phase of the reproductive cycle and after exposure to seminal extracellular vesicles suggests that Tregs in the uterus and vagina may facilitate early pregnancy through localized immunosuppression (219–223). Growing interest in Treg-based therapies highlights their potential to address pregnancy-related complications. However, further clinical research is needed to develop diagnostic tools for early pregnancy Treg profiling and to identify optimal therapeutic windows (224).

## Cervical and vaginal Tregs

The healthy vaginal ecosystem is usually dominated by *Lactobacillus* species, which help prevent colonization by harmful bacteria and fungi. Disruption of this balance (dysbiosis) can cause overgrowth of damaging microorganisms, leading to bacterial vaginosis, candidiasis, urinary tract infections, and a disrupted vaginal pH—factors that collectively increase the risk of STIs and infertility (225, 226). Like the gastrointestinal system, vaginal immunity must tolerate beneficial microbes while remaining able to respond properly to infections. Growing evidence shows that Tregs support this balance. For example, *Lactobacillus crispatus*, a common commensal species, has been shown to induce Treg differentiation from naive CD4^+^ T cells *in vitro* (227). Additionally, microbial imbalance is often linked with increased inflammatory cytokines and decreased Treg levels in peripheral blood, suggesting that loss of microbial harmony may shift immune responses from regulation to inflammation (226, 228, 229). These findings highlight the need for further research on how vaginal Tregs mediate the balance between tolerance and immunity based on microbial signals. Tregs in the uterine and vaginal mucosa not only support homeostasis and pregnancy but also help in immune responses to infections. In mouse models of vaginal HSV-2 infection, Tregs in the draining lymph nodes are crucial for proper movement of antigen-presenting dendritic cells from the infection site. When these Tregs are absent, virus-specific immune responses are delayed, emphasizing their vital role in antiviral defense (46, 214).

## Future prospective

The increasing focus on the immune system’s role in tissue repair and regeneration reflects a shift in regenerative medicine. However, a significant knowledge gap remains about how the adaptive immune system—especially Treg cells—affects tissue healing. Important questions include whether neoantigens produced during injury can trigger specific repair responses, and how T cells distinguish between self- and non-self-antigens in this process. The mechanisms behind T cell recruitment, activation, and function during tissue injury are still not fully understood. To address these issues, future research should utilize advanced models such as antigen tracing in transgenic reporter mice and mass spectrometry to discover neoantigens specific to injury that may induce Treg recruitment and activation. At the same time, combining lineage tracing with single-cell transcriptomics can reveal Treg clonal behavior and antigen specificity in their local environments, shedding light on how individual clones contribute to repair in specific tissues. Progress in immune-based regenerative therapies is also slowed by the lack of specific markers for immune cell subsets involved in tissue repair. Advances in single-cell genomics and multi-omics profiling may identify lineage- and function-specific markers that differentiate reparative immune cells from non-reparative ones. Additionally, the difference between tissues that regenerate without scarring (such as bone) and those prone to fibrosis (like the heart and liver) remains unclear. Gaining a deeper understanding of how immune cells, tissue-resident progenitors, and myofibroblasts interact could reveal mechanisms that promote regeneration over fibrosis and help develop targeted repair strategies.

Another important aspect is the impact of aging on immune-mediated tissue repair. The decline in immune function with age may impair Treg recruitment, function, or stability, ultimately reducing regenerative capacity in older individuals. However, most current studies on Treg function in tissue repair rely on the Foxp3DTR mouse model, which has limitations due to the development of systemic autoimmunity with prolonged Treg depletion. To address this, future studies should include gain-of-function approaches, such as adoptive transfer of purified Tregs into Rag1^+^/^+^ mice, to assess Treg function without the confounding effects of autoimmunity. Emerging conditional genetic models, like Foxp3-CreERT2 mice, offer better temporal control over Treg depletion and avoid long-term systemic effects. When combined with adoptive transfer into lymphopenic hosts, these models enable precise examination of Treg stability, plasticity, and reparative functions over time *in vivo*. These tools are crucial for moving beyond correlative findings toward mechanistic understanding. From a therapeutic standpoint, modulating tissue-resident Tregs to enhance local repair is a promising strategy. Treg populations in tissues such as the lung, skeletal muscle, and skin help maintain local immune homeostasis. Notably, IL-33 signaling expands tissue-resident Tregs and boosts their reparative ability, partly through the induction of AREG, a key factor in tissue repair. However, the full functional diversity among these Tregs and their direct roles in regeneration are still not fully understood.

To translate preclinical findings into human applications, advanced organoid systems and humanized mouse models serve as powerful platforms. Organoids derived from lung, skin, and gut tissues that include immune components can mimic tissue-specific microenvironments, enabling detailed mechanistic and therapeutic screening. Similarly, humanized mice with reconstituted human immune systems provide an *in vivo* environment to study human Treg trafficking, stability, and antigen specificity during tissue injury and repair. Despite their therapeutic potential, Treg-based regenerative treatments encounter key obstacles. These include maintaining the phenotypic stability of Tregs after transfer, ensuring effective trafficking and retention in injured tissues, and selecting antigen-specific Tregs to prevent broad immunosuppression. Overcoming these hurdles is essential for fully realizing the therapeutic potential of Treg-based strategies for tissue repair and regeneration.
